# RCAN1-mediated calcineurin inhibition as a target for cancer therapy

**DOI:** 10.1186/s10020-022-00492-7

**Published:** 2022-06-18

**Authors:** Mengyi Lao, Xiaozhen Zhang, Hanshen Yang, Xueli Bai, Tingbo Liang

**Affiliations:** 1 Department of Hepatobiliary and Pancreatic Surgery, The First Affiliated Hospital, School of Medicine, Zhejiang University, 79 Qingchun Road, Hangzhou, 310009 Zhejiang China; 2 Zhejiang Provincial Key Laboratory of Pancreatic Disease, The First Affiliated Hospital, School of Medicine, Zhejiang University, Hangzhou, 310009 Zhejiang China; 3 Zhejiang Provincial Innovation Center for the Study of Pancreatic Diseases, Hangzhou, 310009 Zhejiang China; 4 Zhejiang Provincial Clinical Research Center for the Study of Hepatobiliary and Pancreatic Diseases, Hangzhou, 310009 Zhejiang China; 5 Cancer Center, Zhejiang University, Hangzhou, 310058 Zhejiang China

**Keywords:** RCAN1, Calcineurin, Cancer therapy

## Abstract

Cancer is the leading cause of mortality worldwide. Regulator of calcineurin 1 (RCAN1), as a patent endogenous inhibitor of calcineurin, plays crucial roles in the pathogenesis of cancers. Except for hypopharyngeal and laryngopharynx cancer, high expression of RCAN1 inhibits tumor progression. Molecular antitumor functions of RCAN1 are largely dependent on calcineurin. In this review, we highlight current research on RCAN1 characteristics, and the interaction between RCAN1 and calcineurin. Moreover, the dysregulation of RCAN1 in various cancers is reviewed, and the potential of targeting RCAN1 as a new therapeutic approach is discussed.

## Background

Currently, cancer is one of the main causes of death globally, and the cancer-associated morbidity and mortality rates have been rapidly increasing (Bray et al. 2021). Worldwide, 19.3 million new cases of cancer and 10 million cancer-related deaths were reported in 2020 (Sung et al. 2020; Ferlay et al. 2020). Molecularly targeted therapy has achieved demonstrable benefits in the treatment of cancer. Elucidating the underlying molecular mechanisms of cancer is essential for its prevention and treatment.

Down syndrome is a common human hereditary disorder that results from full or partial trisomy of chromosome 21 (HSA21). It causes a number of typical deformities and congenital or acquired medical problems. Interestingly, epidemiological studies have demonstrated that Down syndrome is associated with a considerably decreased risk of nearly all solid tumors and a high risk of leukemia, and this has led to investigations into possible solid tumor suppressor genes on HSA21 (Hasle et al. 2000, 2016; Satgé et al. 1998; Patja et al. 2006). Fuentes et al. first identified a new gene, Down syndrome critical region 1 (DSCR1), in a minimal region that is related to the Down syndrome phenotype (Fuentes et al. 1995, 1997a). After the major function of DSCR1 was found to involve modulation of calcineurin (CN) activity under physiological and pathological conditions, DSCR1 was termed regulator of calcineurin 1 (RCAN1) (Davies et al. 2007). RCAN1 has been identified as an endogenous inhibitor of the serine (Ser)/threonine (Thr) phosphatase CN, which has a critical function in the control of nuclear factor of activated T cell (NFAT) dephosphorylation and NFAT nuclear translocation (Aramburu et al. 2000; Fuentes et al. 2000).

Recently, accumulating evidence has indicated that RCAN1 is closely involved in the development of cancer and may be a potential therapeutic target. Therefore, the functions of RCAN1 in cancer pathogenic pathways and its potential as a novel therapeutic target need to be explored. Here, we have summarized recent studies about the structure, function, and regulation of RCAN1, and their association with various types of cancers. In particular, the inhibitory effect of RCAN1 on the CN–NFAT pathway, alone or synergistically with other agents, has been explored in order to shed light on potential treatments for cancer. Thus, the present review explores the functions of RCAN1 in cancer pathogenesis and its potential application in the treatment of cancer.

### RCAN1 structure, function, and regulation

#### RCAN1 gene and protein structure

**Fig. 1:**
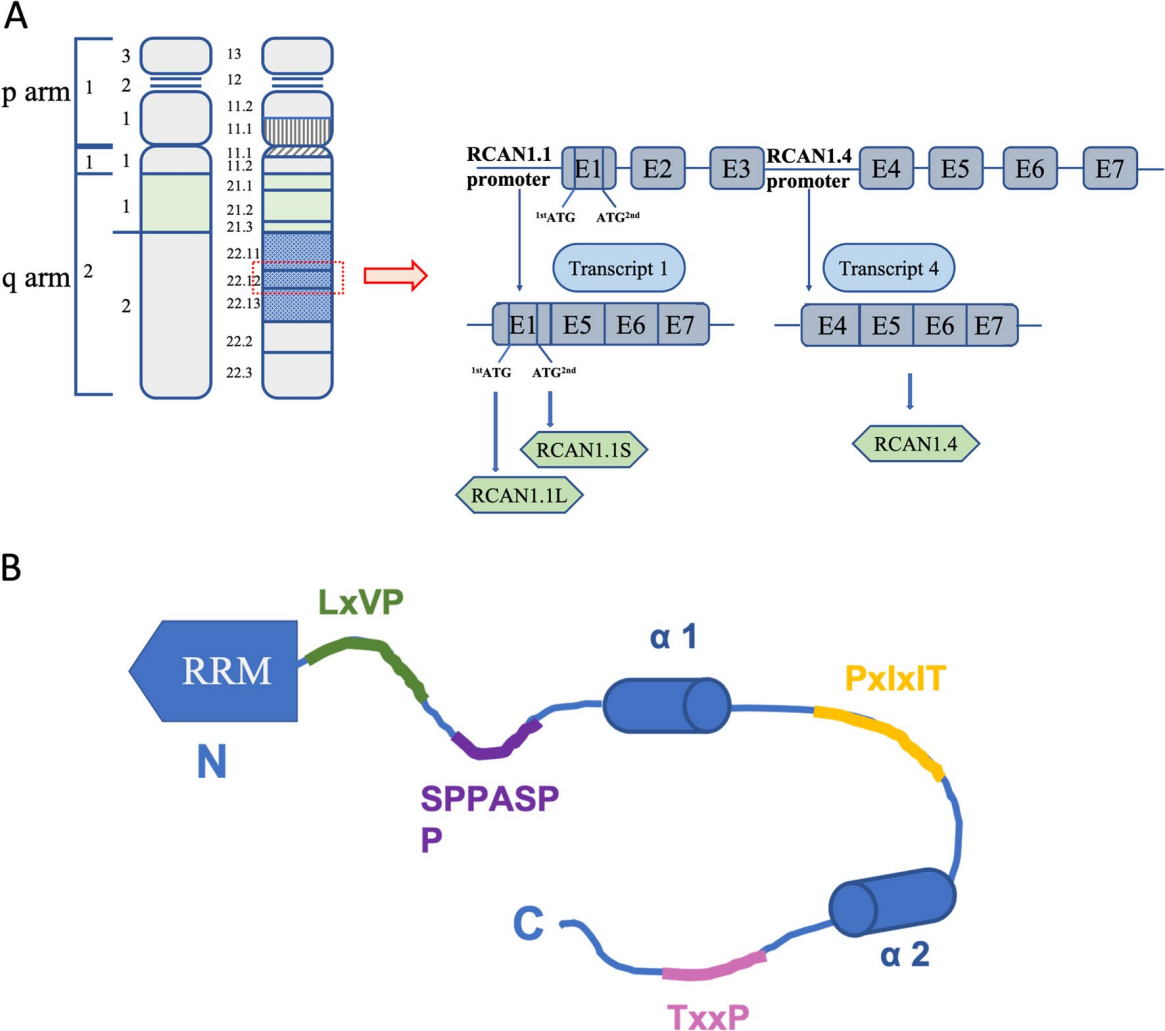
RCAN1 gene and protein structure. **A** A schematic representation of the location of RCAN1 on human chromosome 21. RCAN1 is at location 21q22.12. Gene consists of seven exons (exon 1 to exon 7). By alternative mRNA splicing, two major transcripts, RCAN1.1 (E1, E5–E7) and RCAN1.4 (E4, E5–E7) are generated. By alternative usage of two in-frame translational start codons (presented as 1st ATG and 2nd ATG), two versions of RCAN1.1, RCAN1.1L (252 amino acids) and RCAN1.1S (197 amino acids), are generated. **B** RCAN1 contains a structured N-terminal RNA- recognition motif (RRM) domain, binding mRNA, and a C-terminal domain, binding CN. The C-terminal domain contains LxVP, SPPASPP, PxIxIT, and TxxP motifs, which bind directly to CN

Human RCAN1 gene, identified by coding sequences in 1995, is located on the human chromosome 21q22.12 and consists of seven exons (exon 1 to exon 7) and six introns (Fuentes et al. 1995, 1997a, b, 1999, 2000). Alternative mRNA splicing generates different transcripts that generate two isoforms of RCAN1 in human tissues: RCAN1.1 (transcript 1; exon 1 and exon 5 to exon 7) and RCAN1.4 (transcript 4; exon 4 to exon 7) (Fuentes et al. 1997a). RCAN1 is abundant in many different tissues: for example, high expression of RCAN1.1 has been detected in the brain, heart, and skeletal muscle, and RCAN1.4 expression has mostly been detected in the heart and skeletal muscle (Crawford et al. 1997). The replacement of the start codon of RCAN1.1 with two different in-frame translational start codons results in the generation of two versions of RCAN1.1, namely, RCAN1.1L and RCAN1.1S. RCAN1.1L is the major isoform, and it includes a new up-stream in-frame AUG codon in the optimal context of transcript 1, encoding a 252-amino acid protein. RCAN1.1S is a truncated form of the original protein RCAN1.1 L, encoding a 197-amino acid protein, which has extremely low expression (Fig. 1A) (Genescà et al. 2003; Wu and Song 2013). Different RCAN1 isoforms may have distinct biological activities. RCAN1.4 overexpression can inhibit the CN-NFAT-dependent pathway, including VEGF-induced angiogenic responses, to suppress tumor growth and angiogenesis. However, RCAN1.1L had the opposite effect. Additionally, RCAN1.1 plays a typical role in apoptosis (Wu and Song 2013). RCAN1 plays an important role in protecting cells from hypoxia-related cell death. In hypoxic conditions, RCAN1.1L specifically induces mitophagy and efficient mitochondrial degradation, which in turn contributes to cell survival (Sun et al. 2014).

Transcriptional regulation and signal transduction of RCAN1 is based on an amino acid sequence containing many acidic and proline-rich domains. RCAN1 commonly contains 197 amino acids and includes two domains, a structured N-terminal RNA-recognition motif (RRM) and a C-terminal domain. The former (which comprises amino acids 1 to 88) is involved in binding mRNA, and the latter (which comprises amino acids 89 to 197) is intrinsically disordered as a result of two partially populated α-helices, binding CN and inhibiting its activity (Li et al. 2020). RCAN1 contains eight Ser and six Thr residues, some of which are potential phosphorylation sites, including Ser^93^, Ser^94^, Ser^108^, Ser^112^, Thr^124^, Ser^136^, Thr^153^, Ser^163^, Thr^186^, and Thr^192(^Jung et al. 2011; Pan et al. 2009; Hornbeck et al. 2004). Phosphorylated RCAN1 negatively affects CN binding and reduces its ability to inhibit CN (Fig. 1B) (Abbasi et al. 2006; Lee et al. 2008).

### RCAN1-mediated regulation of CN

CN is a calcium/calmodulin-dependent Ser/Thr phosphatase that comprises a catalytic subunit, namely, calcineurin A (CNA), and a regulatory subunit, namely, calcineurin B (CNB), and is found in several human tissues and functions in many physiological and pathological processes(Klee et al. 1979; Rusnak and Mertz 2000). Calcium-activated CN is involved in the dephosphorylation of several substrates, including NFATs, FOXO (forkhead transcription factors), MEF2 (myocyte-specific enhancer factor 2), and TFEB (transcription factor EB) (Creamer 2020). The NFATs family are the most studied substrates of CN (Klee et al. 1979; Rusnak and Mertz 2000). In colorectal cancers, CN protein expression and its activity were increased compared with those in the corresponding normal mucosae (Masuo et al. 2009). In normal brain, CNA shows little or no expression in astrocytes, but its expression considerably increases in grade IV astrocytoma, especially in areas of high infiltration/migration (Brun et al. 2013). Increased expression of CNA is associated with the development and progression of breast cancer (Masaki et al. 2021). In addition, calcineurin activation plays an essential role in T-cell leukemogenesis (Medyouf et al. 2007). NFAT-mediated Ca^2+^/calcineurin signaling may influence central aspects of tumor biology (Buchholz and Ellenrieder 2007). The deregulation of CN-NFAT signaling is a key part in the development and progression of several tumors, including ovarian cancer, liver cancer, prostate cancer, lymphomas, breast cancer, pancreatic cancer, and lung cancer (Masaki 2022).

**Fig. 2:**
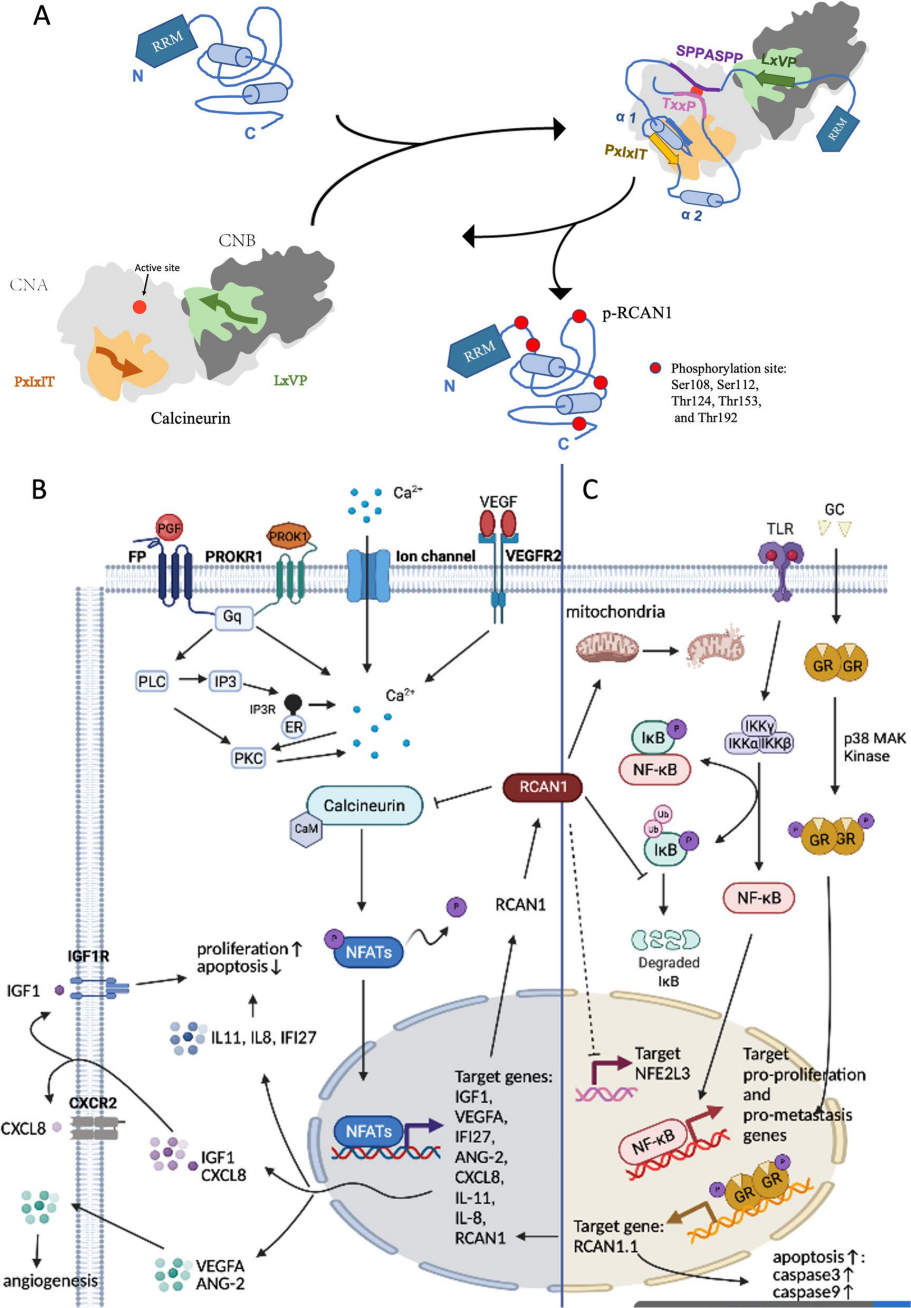
The signaling pathway of RCAN1 in tumor progression. **A** RCAN1 inhibits CN activity by blocking the CN-essential substrate recruitment site, LxVP and PxIxIT interaction grooves, by using its LxVP and PxIxIT motif. The inhibition of CN by RCAN1 can be weakened by phosphorylation of RCAN1. **B** RCAN1-regulated CN. RCAN1 inhibits CN-NFAT pathway and NFAT-regulated gene transcription, including IGF-1, VEGFA, ANG-2, IFI27, CXCL8, IL-8, and IL-11. **C** CN-independent RCAN1 regulation. GC binds to GREs in the RCAN1.1 transcription start site, inducing apoptosis cells. RCAN1 inhibits NFE2L3 and the NF-κB signaling pathway

RCAN1, an inhibitor of CN dephosphorylation, impedes dephosphorylation of many different important physiological substrates of CN, including ion channels and transporters, mitochondrial function regulators, and NFATs (Harris et al. 2005; Roy 2020). Dysregulation or dysfunction of RCAN1 and CN is closely related to carcinogenesis. RCAN1 inhibits CN activity by blocking the essential substrate recruitment site and active site of CN. CN inhibition by RCAN1 can be weakened by phosphorylation of RCAN1 (Li et al. 2020). RCAN1 contains the LxVP motif (amino acids 96–99), SPPASPP motif (amino acids 108–114), PxIxIT motif (amino acids 154–159), and TxxP motif (amino acids 186–189), which bind directly to CN (Li et al. 2020). The PxIxIT motif and the LxVP motif are two short linear motifs (SLiMs) that bind to the PxIxIT- and LxVP-binding regions of CN, respectively (Li et al. 2020, 2007; Grigoriu et al. 2013). The LxVP-binding region is a hydrophobic cleft at the interface of the A and B subunits of CNA (Grigoriu et al. 2013), but this only minimally contributes to CN binding. The PxIxIT-binding region is the catalytic domain of CNA (Li et al. 2007). The ability of PxIxIT for binding CN is 50 times that of the LxVP motif, and this make it the main CN-binding domain of RCAN1 (Li et al. 2020). It plays a critical role in preventing CN from recruiting substrates, such as NFATs (Li et al. 2020; Hendus-Altenburger et al. 2019). The ^186^TxxP^189^ motif and ^108^SPPASPP^114^ motif of RCAN1 (which mimic the known dephosphorylation sites in NFAT) can directly block the active site of CN, thereby reducing the activity of CN against its endogenous substrates (Vega et al. 2002). The CN-binding PxIxIT motif of RCAN1 forms a stable, tertiary domain. When RCAN1 binds to CN, the entire core of RCAN1 folds up and augments additional protein stabilization; this phenomenon is specific to RCAN1 and has not been detected in other regulatory proteins of CN (Li et al. 2020). In addition, RCAN1 contains multiple potential phosphorylation sites. Li et al. determined that the binding ability of phosphorylated RCAN1 (pRCAN1) to CN is 30 times weaker than that of RCAN1, and the phosphorylation sites are Ser^108^, Ser^112^, Thr^124^, Thr^153^, and Thr^192^. For example, phosphorylation of Thr^153^ and Ser^108^ exerts decisive effects on relieving the RCAN1:CN complex and increasing CN activity. Phosphorylation of RCAN1 at Thr^153^ plays an important role in lowering the binding affinity between RCAN1 and CN. Phosphorylation at Ser^108^ reduces RCAN1-mediated inhibition by blocking the catalytic domain and, thereby, suppressing the binding ability of the SPPASPP pseudosubstrate motif (Li et al. 2020). In addition, phosphorylation at Ser^136^ weakens the binding between RCAN1 and CN and promotes CN-NFAT signaling (Liu et al. 2009). pRCAN1 is a CN substrate, which can be dephosphorylated by CN. LxVP motifs are optimally positioned phosphorylation sites (phosphosites) for rapid dephosphorylation by CN, facilitating dephosphorylation of specific substrate residues. In contrast, TxxP motifs act on limiting dephosphorylation of the active site (Li et al. 2020). It is well known that NFAT is the substrate of CN. A sequence similar to NFAT was identified in RCAN1.4, which is also dephosphorylated via CN (Fig. 2A) (Vega et al. 2002).

The mechanism of RCAN1 regulation of CN activity in cancer is complex. In different cell lines, differences in RCAN1 subtypes, phosphorylation sites, and phosphorylation levels are responsible for differences in the effects of RCAN1 on CN (Wu et al. 2014). Importantly, low levels of RCAN1 are required for maintaining CN-NFAT activity. The biphasic role of RCAN1 to CN:RCAN1 functions as a facilitator when its levels are low, but as an inhibitor when its levels are high (Kingsbury and Cunningham 2000; Hilioti et al. 2004; Shin et al. 2011).

Many studies have confirmed that RCAN1 can inhibit cancer cell proliferation and migration via inhibition of the CN-NFAT pathway and NFAT-regulated transcription of genes such as insulin-like growth factor 1 (IGF-1) (Jin et al. 2017), interferon alpha inducible protein 27 (IFI27) (Lao et al. 2021), C-X-C motif chemokine ligand 8 (CXCL8), interleukin-8 (IL-8), and interleukin-11 (IL-11) (Sales et al. 2009, 2010; Maldonado-Pérez et al. 2009). In addition, a single extra transgenic copy of RCAN1 in RCAN1 transgenic mice is sufficient to significantly suppress tumor growth, which results from resisting tumor angiogenesis via suppression of the calcineurin pathway (Baek et al. 2009). However, Ryeom et al. reported that the deletion of RCAN1 leads to hyperactivated CN and precocious endothelial apoptosis, suppressing tumor growth and angiogenesis (Ryeom et al. 2008). Accumulating evidence indicates that RCAN1.4 inhibits endothelial cell migration and angiogenesis via CN-NFAT signaling (Ryeom et al. 2008; Minami et al. 2004; Yao and Duh 2004). Vascular endothelial growth factor A (VEGFA) functions downstream of the CN-NFAT pathway, participating in RCAN1.4 inhibition of endothelial cell proliferation and angiogenesis (Jin et al. 2017; Lao et al. 2021). While overexpression of RCAN1.1 may promote angiogenesis (Ryeom et al. 2008). Moreover, enhanced RCAN1 levels increased antitumor drug, lenalidomide (LEN), sensitivity by mediating CN-NFAT activity (He et al. 2020). Together, RCAN1 binds to CN and inhibits CN activity, preventing different cancers via dephosphorylation of antitumor substrates (Fig. 2B).

### CN-independent functions of RCAN1

Not all functions of RCAN1 are dependent on CN as CN-independent functions of RCAN1 have been reported. Firstly, previous studies have shown that increased RCAN1 overexpression promotes apoptosis by mediating caspase-3/caspase-9 expression, which is independent of CN and is a possible mechanism for inhibiting tumor growth (Wu and Song 2013; Sun et al. 2011; Fu and Wu 2018). RCAN1 overexpression inhibits cytochrome *c* release from mitochondria, which is necessary to activate caspase-9. Activated caspase-9 recruits and activates its downstream executor, caspase-3, which subsequently cleaves its substrates and initiates the apoptotic pathway (Sun et al. 2011). In addition, glucocorticoid (GC) binds to glucocorticoid response elements (GREs) in the RCAN1.1 transcription start site, inducing apoptosis in cells (Saenz et al. 2015; Hirakawa et al. 2009). Secondly, RCAN1 is identified as an endogenous inhibitor of the NF-κB pathway, which is important for the modulation of cell survival, growth, and differentiation (Liu et al. 2015a; Yang et al. 2009; García-Redondo et al. 2018; Pang et al. 2018; Pacifico and Leonardi 2006; Ben-Neriah and Karin 2011; Gerondakis et al. 1999). The *N*-terminal amino acids 1–103 of RCAN1 physically interact with IκBα, an NF-κB inhibitory protein, and affect its tyrosine 42 phosphorylation to inhibit its self-degradation, which may dampen NF-κB activity (Liu et al. 2015b).

Notably, unlike hypoxia-induced downregulation of RCAN1.4, which activates the CN-NFAT signaling pathway (Huang et al. 2020; Kim et al. 2020), RCAN1-1L overexpression protects against hypoxia-induced apoptosis and contributes to cell survival in hypoxic conditions by induction of mitophagy, partly through Parkin (Sun et al. 2014; Yan et al. 2014). RCAN1.1L overexpression induces mitochondrial autophagy (Pang et al. 2018, Pacifico and Leonardi 2006), and high glucose (HG)-induced RCAN1.4 upregulation increases mitochondrial fragmentation (Duan et al. 2015; Ermak et al. 2012; Chen et al. 2020). In addition, RCAN1 can help to overcome the stress and enhance survival under endoplasmic reticulum (ER) stress conditions (Bartoszewski et al. 2020; Belmont et al. 2008). Moreover, RCAN1.4 inhibits tumor-promoting gene, erythroid 2-like 3 (NFE2L3), in thyroid cancer and alleviates the inhibition of sunitinib in sunitinib-resistant clear cell renal cell carcinoma (ccRCC) (Song et al. 2018; Wang et al. 2017) (Fig. 2C).

### Regulation of RCAN1

The regulation of RCAN1 at the transcriptional, translational, and post-translational level is described below.

**Fig. 3:**
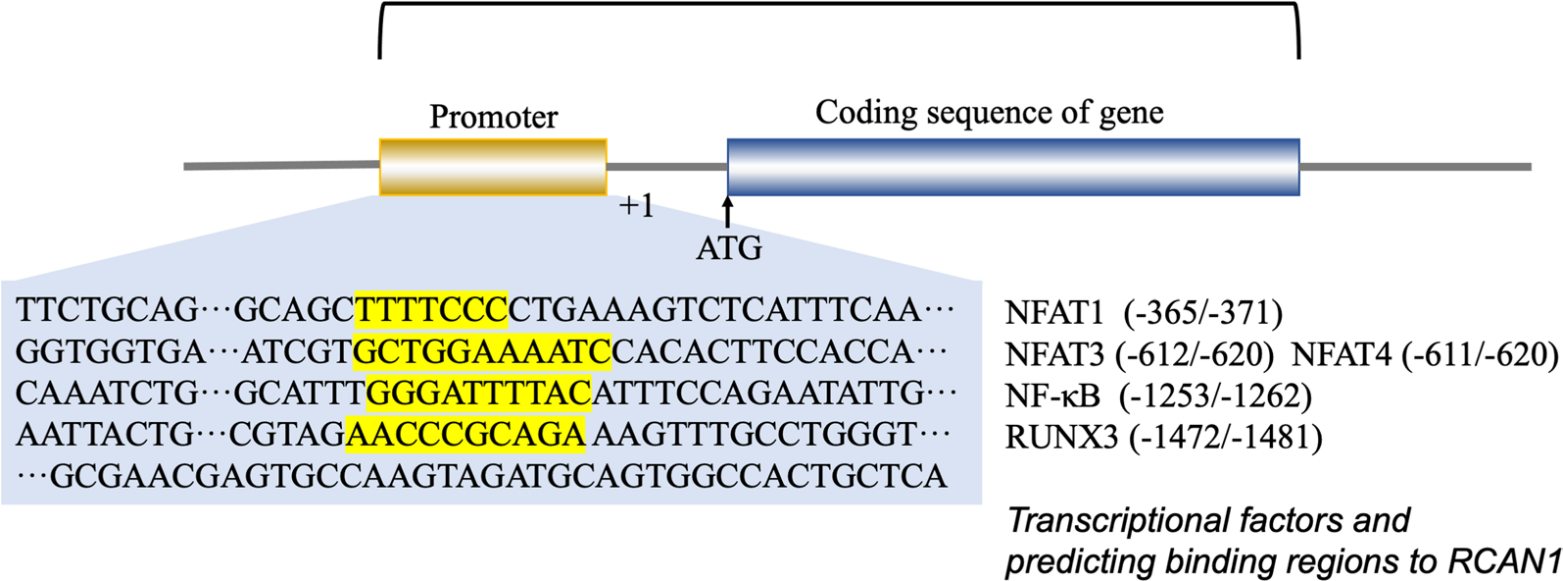
Transcription factors and their predicted binding regions in RCAN1. The promoter sequence of RCAN1 and a list of transcription factors and their predicted binding regions in RCAN1

Transcriptional regulation is mediated via the promoter region present upstream of RCAN1.4, which contains 15 potential NFAT-binding sites. RCAN1 gene transcription is suppressed by NFAT protein expression via a negative feedback loop (Li et al. 2020; Yang et al. 2000). Consistently, an NF-κB responsive element was identified in the 576–554 bp region of the RCAN1 promoter. Other studies have shown that the expression of RCAN1 is enhanced by activation of the NF-κB pathway (Lee et al. 2008; Fang et al. 2019; Zheng et al. 2014). In addition, the RCAN1.1 promoter region contains a functional GRE, and RCAN1.1 is upregulated by glucocorticoids at the transcriptional level (Yang et al. 2000; Nagao et al. 2012). Some tumor-derived exosome (TEX) miRNAs can promote angiogenesis and metastasis of tumors by inhibiting RCAN1 (Kim et al. 2020; Zheng et al. 2021; Zhang et al. 2020). Super-enhancers (SEs) are defined as large clusters of transcriptional enhancers and are found in the RCAN1.4 sequences. For example, RCAN1.4-SE^distal^ was detected about 266 kb downstream of RCAN1.4. SEs are known to promote the expression of genes that play a role in cell identity (Hnisz et al. 2013). The SE region of RCAN1.4 contains binding sites that are consistent with the sequences of the transcription factor RUNX3. Thus, normal RUNX3-mediated SE-driven RCAN1.4 expression can suppress tumorigenesis (Deng et al. 2020). Transcription factor ATF6 can induce RCAN1 expression. ER stress can activate the unfolded protein response and transcription factor ATF6. RCAN1, an ATF6-inducible gene, can decrease the need for ER protein folding, thus helping to overcome the stress and enhance survival under ER stress conditions (Bartoszewski et al. 2020; Belmont et al. 2008). Figure 3 shows the promoter sequence of RCAN1 and lists transcription factors and their predicted binding sites on RCAN1.

In translational regulation, RCAN1.1 has two isoforms, RCAN1.1S and RCAN1.1L, which result from altering the translational start codons. Furthermore, RCAN 1.1S expression is considerably lower than RCAN 1.1L expression; this is a result of differences in the efficiency of translation initiation of the in-frame AUG sites (Genescà et al. 2003; Wu and Song 2013). Different RCAN1.1 isoforms may have distinct cellular functions. For example, RCAN1.1L and RCAN1.1S have an opposite effect on apoptosis. RCAN1.1L overexpression attenuated caspase-3 activation under oxidative stress. However, RCAN1.1S exacerbated the caspase-3 activity increase. Long-term overexpression of RCAN1.1L promotes oxidative stress-induced apoptosis by caspase-3 activation (Wu and Song 2013; Sun et al. 2011). In addition, overexpression of RCAN1.1L can induce NFAT activation, and promote proliferation of endothelial cells and angiogenesis (Qin et al. 2006).

In post-translational regulation, the main post-translational modification of RCAN1 is phosphorylation. pRCAN1 increases CN activity by lowering the affinity of RCAN1 for CN, thus accelerating dissociation of the RCAN1-CN complex (Genescà et al. 2003; Abbasi et al. 2006; Lee et al. 2008). Many kinases and kinase complexes play a role in the phosphorylation of RCAN1, for instance, glycogen synthase kinase 3β (GSK-3β), mitogen-activated protein kinase (MAPK), dual specificity tyrosine phosphorylation-regulated kinase 1A (DYRK1A), p38α MAP kinase, ERK2 (Vega et al. 2002), TGF-β activated kinase 1 (TAK1)–TAK1 binding protein 1 (TAB1)–TAK1 binding protein 2 (TAB2) complex, and mitogen-activated protein kinase kinase 3 (MEKK3)–mitogen-activated protein kinase kinase 5 (MEK5)–big mitogen-activated protein kinase 1 (BMK1) complex (Jung et al. 2011; Abbasi et al. 2006; Vega et al. 2002; Liu et al. 2009; Ma et al. 2012; Kim et al. 2012). However, mitogen-activated protein kinase kinase kinase 14 (NIK), protein kinase A (PKA), and DYRK1A phosphorylate RCAN1 to enhance RCAN1’s ability to inhibit the CN-NFAT pathway. The molecules that phosphorylate RCAN1, their preferred phosphorylation sites, and their effects on RCAN1 activity are summarized in Table 1.

**Table 1:** The molecules of phosphorylated RCAN1

The effects on RCAN1 activity	Molecules of phosphorylate RCAN1	Phosphorylation site	References
Accelerate dissociation of the RCAN1-CN complex and increases CN activity	MEK5-BMK1-MEKK3 complex	Serine-108 and serine-112	Abbasi et al. (2006); Abbasi et al. (2005)
	TAK1–TAB1–TAB2 complex	Serine-94 or serine-136 sites	Liu et al. (2009)
	MAPK	Serine-112	Vega et al. (2002)
	GSK-3β	Serine-108	Jung et al. (2011)
	ERK2	Serine-112	Vega et al. (2002)
	P38MAPK	Serine-93, -108, -112; threonine-124, -153	Ma et al. (2012)
Enhance RCAN1’s ability to inhibit the CN-NFAT pathway	NIK	–	Lee et al. (2008)
	DYRK1A	Serine-112 and threonine-192	Smith (1998)
	PKA	–	Kim et al. (2012)

**Table 2:** The role of RCAN1 in various tumors

Cancer types	Tumor tissue expression: down-or upregulated	Function: Inhibition or promotion	How to regulate RCAN1	The specific signaling pathways downstream of RCAN1	Clinical significance	References
Breast cancer	Downregulated	Inhibition	RUNX3 resulted in a decreased RCAN 1.4 expression; oxytocin induced nuclear translocation of NFAT to induce expression of RCAN1	RCAN1.4 Inhibited CN-NFAT2 signaling	–	Deng et al. (2020); Behtaji et al. (2021)
Lung cancer	Downregulated	Inhibition	Hypoxia induced the release of specific exosome miR-619-5p and inhibited RCAN1.4	Modulated the level of VEGF-VEGFA; blocking of CN—NFAT signaling and CN-NFAT—angiopoietin-2 signaling axis	Arsenic trioxide (As2O3) inhibited SCLC metastasis by upregulating RCAN1	Baek et al. (2009); Kim et al. (2020); Shin et al. (2014); Minami et al. (2013); Ma et al. (2017); Zheng et al. (2019)
Hepatocellular carcinoma	Downregulated	Inhibition	miRNAs (miR-877, miR‐572 and miR-182-5p) negatively regulated RCAN1; aberrant CpG methylation in the 5’ regulatory region of RCAN1.4 induced RCAN1.4 down-regulation	RCAN1.4 inhibited IGF-1 and VEGFA by inhibiting CN—NFAT1 signaling	–	Jin et al. (2017); Song et al. (2019); Shi et al. (2018)
Pancreatic carcinoma	Downregulated	Inhibition	–	RCAN1.4 inhibited IFI27 and VEGFA by inhibiting CN—NFAT1 signaling	–	Lao et al. (2021); Lee et al. (2013)
Colorectal carcinoma	Downregulated	Inhibition (Targeted RCAN1 deletion suppresses tumor growth)	PPARγ positively regulated RCAN1	Inhibited CN-NFAT signaling pathway	As a biomarker to predict recurrence in stages II and III of colon cancer	Ryeom et al. (2008); Espinosa et al. (2009); Sebio et al. (2015); Bush et al. (2007)
Renal carcinoma	Downregulated	Inhibition (Targeted RCAN1 deletion suppresses tumor growth)	–	Regulated VEGF-CN-NFAT signaling	RCAN1.4 reduced sunitinib resistance	Ryeom et al. (2008); Song et al. (2018)
Bladder cancer	Downregulated	–	–	–	The level of RCAN1 expression in urine enabled diagnosis of bladder cancer	Eissa et al. (2019)
Endometrial adenocarcinoma	Downregulated	Inhibition	–	Inhibited CN-NFAT signaling pathway to negatively regulate the expression of CXCL8, IL-8, and IL-11	–	Sales et al. (2009); Maldonado-Pérez et al. (2009); Sales et al. (2010)
Epithelial ovarian cancer	–	Not significantly affected	–	–	–	Hata et al. (2009)
Thyroid cancer	Downregulated	Inhibition	Metastin enhanced the expression of RCAN1	Inhibited NFE2L3 and CN-NFAT signaling	–	Wang et al. (2017); Espinosa et al. (2009); Stathatos et al. (2005))
Oral squamous cell carcinoma	Downregulated	Inhibition	miR-103a-3p downregulated RCAN1	–	–	Zhang et al. (2020)
Hypopharyngeal and Laryngopharynx cancer	Upregulated	Promotion	–	Activated VEGF signaling pathway	–	Lv et al. (2017); Lü et al. (2011)
Leukaemia and other hematonosis	–	Inhibition	GC/CR complex induced RCAN1.1	RCAN1.1 inhibited CN activity	RCAN1.1 increased sensitivity to GC and enhanced sensitivity to LEN in MDS/AML	Saenz et al. (2015); Hirakawa et al. (2009); Nagao et al. (2012); Shen et al. 2004)
Lymphoma and Glioma	–	Inhibition	–	Inhibited NF-κB		Liu et al. (2015b); Chen et al. (2017)

## RCAN1 expression, regulation, and function in cancer

### RCAN1 expression in various cancers based on TCGA database

**Fig. 4:**
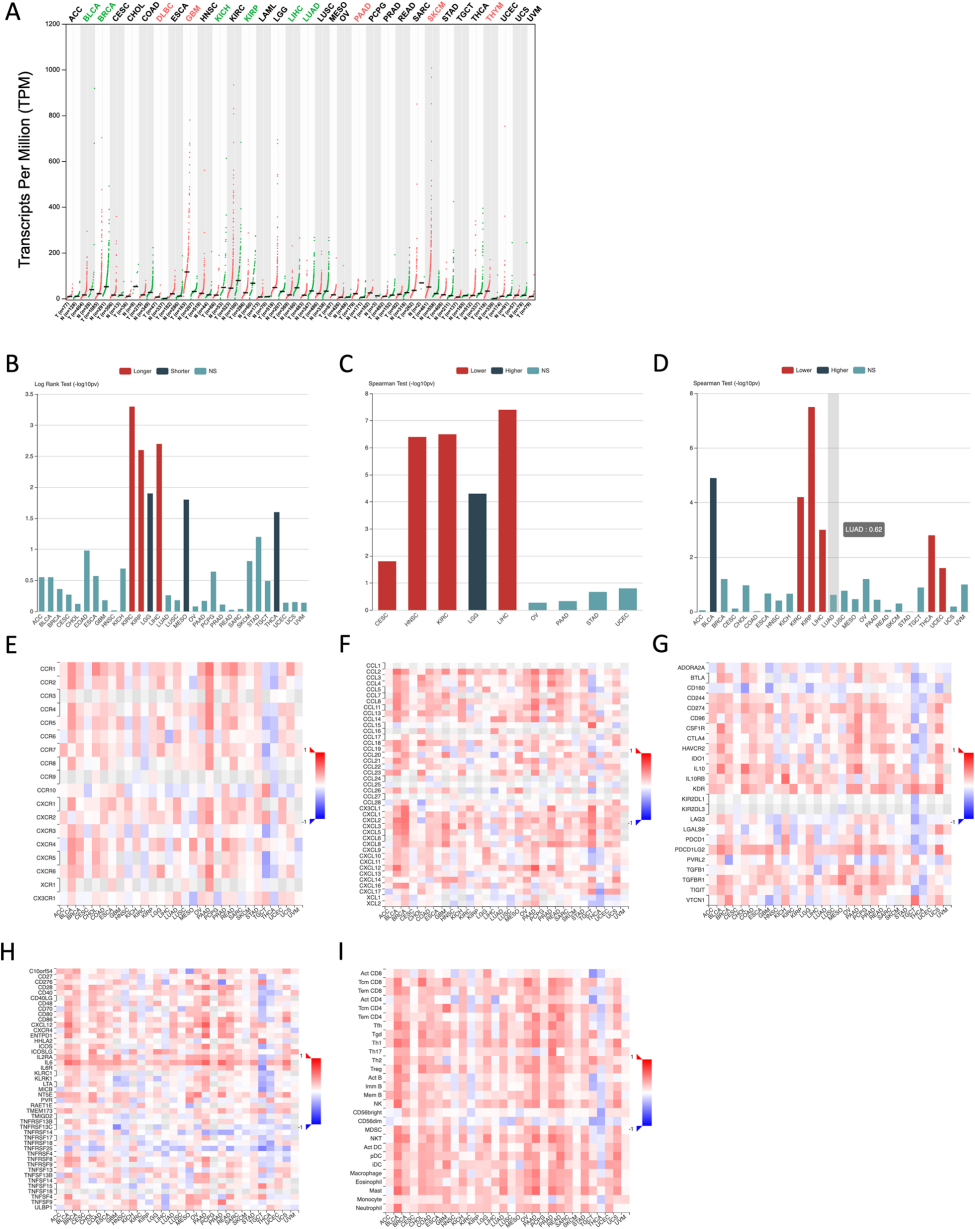
Bioinformatic analysis of RCAN1 in various cancers based on TCGA database. **A** The expression profile of RCAN1 in various cancers. **B** Associations between RCAN1 expression and overall survival across human cancers (x-axis). **C** Associations between RCAN1 expression and grade across human cancers (x-axis). **D** Associations between RCAN1 expression and stage across human cancers (x-axis). **E** Associations between RCAN1 expression and receptors across human cancers (x-axis). **F** Associations between RCAN1 expression and chemokines across human cancers (x-axis). **G** Associations between RCAN1 expression and immunoinhibitors across human cancers (x-axis). **H** Associations between RCAN1 expression and immunostimulators across human cancers (x-axis). **I** Associations between RCAN1 expression and tumor infiltrating lymphocytes (TILs) across human cancers (x-axis)

To determine expression profiles of RCAN1 in various cancers, we examined data on RCAN1 expression in tumors and normal tissues deposited in The Cancer Genome Atlas (TCGA) database. Figure 4A shows that RCAN1 expression is considerably decreased in various cancers, including bladder urothelial carcinoma (BLCA), breast invasive carcinoma (BRCA), kidney chromophobe (KICH), kidney renal papillary cell carcinoma (KIRP), liver hepatocellular carcinoma (LIHC), and lung adenocarcinoma (LUAD). However, in lymphoid neoplasm diffuse large B-cell lymphoma (DLBC), glioblastoma multiforme (GBM), pancreatic adenocarcinoma (PAAD), skin cutaneous melanoma (SKCM), and thymoma (THYM), RCAN1 expression in tumor samples is higher than that in normal samples (Fig. 4B).

### Breast cancer

Down syndrome has been associated with a considerable decrease in the prevalence of breast cancer (Hasle et al. 2000, 2016; Hasle 2001). The RCAN1^BAC−Tg^ mouse line, where RCAN1 expression is moderately higher than expression of the native RCAN1 gene, was generated to explore the biochemistry of RCAN1 and its role in Down syndrome. BAC-Tg mice were crossed with p53^flex7/flex7^; WAP^cre/+^ mice to spontaneously form p53-null mammary epithelial cell adenocarcinomas. However, low levels of RCAN1 overexpression, approximately 1.5-fold higher than the native RCAN1 gene, under in vivo conditions do not result in tumor suppression in the absence of HSA21 genes (Xing et al. 2013). Analysis of breast cancer tissues showed significant downregulation of RCAN1 in comparison with its expression in adjacent non-cancerous tissues from patient samples (Behtaji et al. 2021). RCAN1.4, which was identified as an endogenous tumor suppressor, was found to be closely involved in breast cancer progression and metastasis via inhibition of CN-NFAT2 signaling (Deng et al. 2020). The CN-NFAT pathway is known to be activated in patients with breast cancer and plays an important role in its tumorigenic and metastatic properties (Goshima et al. 2019; Quang et al. 2015; Iampietro et al. 2014; Baggott et al. 2012).

RCAN1 was deemed as an oxytocin-related gene (Behtaji et al. 2021). Oxytocin, which is released from the posterior pituitary, is important for lactation (Young et al. 1996; Fuchs et al. 1991; Nishimori et al. 1996) and the pathogenesis of breast cancer (Behtaji et al. 2021). Oxytocin has been found to induce the transcription and nuclear translocation of endogenous NFAT, as well as CN-NFAT-dependent RCAN1 expression (Pont et al. 2012). The super-enhancer (SE), located *ca*. 266 kb downstream of RCAN1.4, drove RCAN1.4 expression and its tumor inhibitory function. RUNX3 is a transcription factor that plays a role in the regulation of RCAN1.4 expression by the distal super-enhancer. Therefore, the loss of RUNX3 resulted in a decrease in SE-driven RCAN1.4 expression in breast cancer (Deng et al. 2020). Above all, RCAN1 is a tumor suppressor protein, inhibiting tumor growth and tumor angiogenesis in breast cancer.

### Lung cancer

In lung cancer, RCAN1 inhibits tumor proliferation, angiogenesis, and metastases by inhibiting the CN-NFAT pathway. Using mouse models of Down syndrome, the incidence of lung tumors was decreased, in part, because of the suppression of tumor angiogenesis because of attenuated CN signaling in endothelial cells. A similar result was demonstrated in a transgenic mouse model with three copies of the RCAN1 gene using xenografted lung tumors (Baek et al. 2009). LSL-Kras^G12D^ mice carry a single extra copy of RCAN1 to get inducible and more accurately repeatable molecular features of human lung adenocarcinoma in RCAN1 transgenic mouse (Shin et al. 2014). Upregulated RCAN1 contributes to limiting the growth of lung tumors by suppressing lung adenocarcinoma proliferation and angiogenesis and increasing apoptosis through inhibition of the CN pathway (Baek et al. 2009; Shin et al. 2014). Using transgenic mouse models, RCAN1 overexpression inhibits lung metastases, whereas RCAN1 deletion and constitutively activated CN accelerates the formation of lung metastases (Minami et al. 2013). The inhibitory effect of RCAN1 on lung metastases was achieved by modulating the level of VEGF–VEGFA and CN–NFAT–angiopoietin-2 signaling axis of lung endothelium (Minami et al. 2013).

In small cell lung cancer (SCLC), RCAN1 inhibited proliferation, colony formation, migration, invasion, and bone adherence, but not cell apoptosis, in vitro (Ma et al. 2017). The inhibitory effect of arsenic trioxide (As2O3) on SCLC metastasis may be related to the blocking of CN-NFAT signaling by upregulating RCAN1 (Zheng et al. 2019). In non-small cell lung cancer (NSCLC), RCAN1.4 was found to be a target of exosome miR-619-5p. Hypoxic conditions induced NSCLC cells to release miR-619-5p to promote angiogenesis, growth, and metastasis of tumors by targeting the modulation of RCAN1.4. Moreover, significantly lower RCAN1.4 expression and significantly higher miR-619-5p expression in tumor tissues than in normal lung tissues were reported in patients with NSCLC (Kim et al. 2020).

### Gastrointestinal neoplasms

Previous studies demonstrated that RCAN1 played an inhibitory role in the progression of some gastrointestinal cancers, like hepatocellular carcinoma (HCC), pancreatic carcinoma, and colorectal carcinoma.

Numerous studies demonstrated that microRNAs (miRNAs) negatively regulate RNA targets at the post-transcriptional level (Cheng et al. 2015). In HCC, oncogenic miRNAs, including miR-877, miR‐572, and miR-182-5p, negatively regulate RCAN1 to promote HCC progression (Zheng et al. 2021; Song et al. 2019; Shi et al. 2018). Thus, RCAN1 expression was reduced in primary HCC compared to adjacent non-cancer liver tissues (Zheng et al. 2021; Song et al. 2019; Shi et al. 2018). The inhibitory effect of RCAN1 in HCC was further refined to its subtype RCAN1.4 (Jin et al. 2017). RCAN1.4 inhibited expression of IGF-1 and VEGFA by the CN-NFAT1 pathway to suppress the growth, metastasis, and angiogenesis of HCC. And aberrant CpG methylation in the 5’ regulatory region of RCAN1.4 induced RCAN1.4 downregulation in HCC (Jin et al. 2017). In addition, RCAN1 was reduced in a HCC mouse model, by exposure to *N*-nitroso genotoxic hepatocarcinogens, diethylnitrosamine (DEN), and ethylnitrosourea (ENU) (Suenaga et al. 2013; Watanabe et al. 2009), and in a CCl4-induced liver fibrosis mouse model (Pan et al. 2019).

The epidemiological study showed that the prevalence of pancreatic cancer is more than sevenfold lower in patients with Down syndrome than in the general population (Yang et al. 2002). Accordingly, in Pdx-1-Cre; LSL-Kras^G12D^; Dscr1 transgenic mice, RCAN1 seemed to prevent oncogenesis of pancreatic cancer (driven by oncogenic Kras^G12D^; P = 0.1161). Moreover, in pancreatic intraepithelial neoplasia (PanIN), RCAN1 inhibited the progression of early PanIN lesions via attenuation of NFAT and upregulation of the tumor suppressor p15Ink4b, but did not affect the development of PanIN lesions mediated by oncogenic Kras (Lee et al. 2013). In a previous study conducted by our group, RCAN1.4 was found to act as a pancreatic cancer suppressor by blocking the CN-NFAT signaling pathway. IFI27 and VEGFA are important for RCAN1.4-mediated PDAC progression and angiogenesis, respectively (Lao et al. 2021).

In colon cancer cell lines, RCAN1 suppressed tumor growth and metastasis by inhibiting the CN-NFAT signaling pathway (Ryeom et al. 2008; Espinosa et al. 2009). Obesity is a risk factor of colon cancer (CRC) and has also been linked to colon cancer recurrence and survival (Adams et al. 2007; Calle et al. 2003). RCAN1, an obesity-related gene, was regarded as a biomarker for predicting recurrence in stages II and III of colon cancer (Sebio et al. 2015). Peroxisome proliferator-activated receptor γ (PPARγ) was identified as a cancer suppressor in colon carcinogenesis by mouse models (Kohno et al. 2001; Tanaka et al. 2001; Girnun et al. 2002; Osawa et al. 2003; Marin et al. 2006) and clinical samples (Sebio et al. 2015). RCAN1 is regulated by PPARγ in colorectal cancer cells. The novel PPARγ/RCAN1/CN/NFATc axis may play a role in colon carcinogenesis (Bush et al. 2007).

### Urogenital neoplasms

In renal cell carcinoma (RCC), RCAN1.4 restrained tumor progression and metastasis by inhibiting the CN-NFAT signaling pathway (Song et al. 2018). Hypoxia (Jiao and Nan 2012; Joseph et al. 2018) and epithelial–mesenchymal transition (EMT) (Thiery 2003; Kalluri and Weinberg 2009), the hallmarks of the tumor microenvironment, interacted to induce tumor progression and cancer drug resistance. RCAN1.4 relived sunitinib resistance of RCC by negatively regulating the expression of hypoxia inducible factor 2 alpha (HIF2α) and EMT (Song et al. 2018).

Similarly, the levels of RCAN1 mRNA were significantly lower in tumor tissues than in normal tissues in bladder cancer (Eissa et al. 2019). Interestingly, the levels of RCAN1 in urine samples of the bladder cancer group were lower than those in the control group (Eissa et al. 2019).

Chemoattractant cytokines (chemokines), cyclooxygenase (COX) enzymes, and prostaglandins (PGs) play a significant role in uterine pathology. Expression of COX-2, PG, some chemokines (such as IL-8, IL11), and nuclear and membrane-bound G protein-coupled receptors (like the F-prostanoid (FP) receptor) was upregulated in endometrial adenocarcinomas (Sales et al. 2009, 2010; Maldonado-Pérez et al. 2009). The prostaglandin F_2α_-F-prostanoid (PGF_2α_-FP) receptor has been found to promote the growth of endometrial tumors via regulation of vascular function. Activation of PGF_2α_-FP receptor signaling can lead to upregulation of tumorigenic and angiogenic genes such as COX-2, fibroblast growth factor 2, and VEGFA via Gq activation of inositol-1,4,5-trisphosphate (Sales et al. 2009). RCAN1.4, CXCL8, and IL-11 are the targets of PGF_2α_-FP receptor signaling, regulating the CN-NFAT pathway (Sales et al. 2009, 2010). Prokineticin 1 (PROK1), via the PROK1 receptor, induces the expression of RCAN1.4 and IL-8 and regulates the CN-NFAT pathway (Maldonado-Pérez et al. 2009). RCAN1.4, which is known to be an endogenous inhibitor of the CN-NFAT signaling pathway, negatively regulates CXCL8, IL-8, and IL-11 expression (Sales et al. 2009, 2010; Maldonado-Pérez et al. 2009). IL-8, a member of the CXC chemokine family, plays crucial roles in neutrophil chemotaxis/activation and T-cell chemotaxis (Larsen et al. 1989). IL-8 has also been found to play a role in the chemotaxis and proliferation of endothelial cells under in vitro conditions and angiogenesis under in vivo conditions (Koch et al. 1992). CXCL8, a chemokine ligand of the CXCR2 receptor, were implicated in tumorigenesis by enhancing endometrial adenocarcinoma cell (Sales et al. 2009) and melanoma cell (Richmond et al. 1986) proliferation and alveolar epithelial neoplasia (Wislez et al. 2006) and breast cancer (Bieche et al. 2007) development. IL-11/IL-11R expression was associated with proliferation, differentiation, metastasis, and poor prognosis of gastric, breast, and colorectal cancer (Nakayama et al. 2007; Yamazumi et al. 2006; Hanavadi et al. 2006). In epithelial ovarian cancer, the expressions of RCAN1.1, RCAN1.4, and CN-related gene did not play a role in tumorigenesis and prognosis (Hata et al. 2009).

### Head and neck neoplasms

In head and neck neoplasms, RCAN1 played remarkable biphasic roles in regulation of the progressions of thyroid cancer, oral squamous cell carcinoma (OSCC), hypopharyngeal cancer, and laryngopharynx cancer. In papillary thyroid cancer, a loss of RCAN1 expression was found in lymph node metastasis compared to primary tumors (Stathatos et al. 2005). RCAN1 (or RCAN1.4) is a metastasis suppressor, having inhibitory tumor growth and metastasis effects in vitro and in vivo (Wang et al. 2017; Espinosa et al. 2009; Stathatos et al. 2005). RCAN1, along with metastin—another metastasis suppressor, was found to inhibit thyroid cancer metastasis by reducing the activity of CN-NFAT signaling (Espinosa et al. 2009; Stathatos et al. 2005). Metastin, a truncated fragment of *KiSS-1*, acts as a ligand for an orphan G protein-coupled receptor called AXOR12 (Muir et al. 2001; Ohtaki et al. 2001). Metastin/AXOR12 signaling not only suppressed the aggressive tumor phenotype in epithelial ovarian cancer (Hata et al. 2009), melanoma (Shirasaki et al. 2001), thyroid cancer (Ringel et al. 2002), esophageal carcinoma (Sanchez-Carbayo et al. 2003), urinary bladder cancer (Dhar et al. 2004), and gastric carcinoma (Ikeguchi et al. 2004), but also enhanced the expression of RCAN1 (Stathatos et al. 2005). NFE2L3 is involved in these RCAN1.4-mediated inhibitory tumor effects and its overexpression can also independently increase cell invasion (Wang et al. 2017). OSCC had increased microRNA (miR-103a-3p) expression, which was closely correlated to poor prognosis. MiR-103a-3p induced an increase in the proliferation of OSCC cells and inhibited their apoptosis by downregulating RCAN1.

Conversely, in laryngopharynx cancer and hypopharyngeal cancer, RCAN1 expression was significantly higher in cancerous tissues than in peri-cancerous tissues (Lv et al. 2017; Lü et al. 2011). RCAN1 expression was positively correlated with the VEGF-C expression and microvessel density (MVD) in cancerous tissues, which resulted from potent activation of angiogenesis (Lv et al. 2017). The biphasic role of RCAN1, its characteristics as both tumor inhibitor and tumor promoter, mean that RCAN1 is highly expressed in some tumors (Kingsbury and Cunningham 2000; Hilioti et al. 2004; Shin et al. 2011).

### Leukemia and other cancers

Individuals (especially children) with Down syndrome have a greater risk of developing leukemia than the general population (Hasle et al. 2000; Yang et al. 2002). A previous study demonstrated that mutated JAK2 (Janus kinase 2), not dysfunctional RCAN1, may be a common molecular event in leukemia associated with Down syndrome (Bercovich et al. 2008). GC, a major therapeutic agent for leukemia, binds to the GC receptor (GR) inducing apoptosis of leukemia cells from changes in regulatory genes that modulate key pro- and anti-apoptotic genes. RCAN1.1 has a cluster of putative GREs in the transcription start site that are responsible for the response to GC (Saenz et al. 2015; Hirakawa et al. 2009). Therefore, the GC/CR complex can induce RCAN1.1 expression with the help of p38 MAP kinase (Sun et al. 2011; Saenz et al. 2015; Hirakawa et al. 2009; Nagao et al. 2012). In addition, GC-mediated RCAN1.1 upregulation led to downregulation of pro-survival genes by inhibition of CN activity (Hirakawa et al. 2009). E4BP4, a bZIP transcription factor, was upregulated in GC-evoked apoptosis, which facilitates the downstream upregulation of the pro-apoptotic gene, *BIM*, and *RCAN1.1* (Saenz et al. 2015). The overexpression of RCAN1 can increase GC sensitivity to activation by the cAMP response element binding protein (CREB) (Nagao et al. 2012) and ectopic E4BP4 expression (Saenz et al. 2015).

LEN, a therapeutic agent for multiple myeloma (MM) and lower-risk myelodysplastic syndromes (MDS) with lower-risk del(5q) (MDSL) (Pan and Lentzsch 2012), results in a poor response in cases of higher-risk MDS or acute myeloid leukemia (AML) (Fang et al. 2011; Farag et al. 2006). RCAN1 is upregulated in MDSL cells on exposure to LEN. Cyclosporin (Cys), a pharmacological inhibitor of CN, enhanced MDS/AML sensitivity to LEN in vitro (He et al. 2020).

RCAN1 resulted in a decrease in the cell viability of lymphoma Raji cells and inhibited the growth of lymphoma transplants in mice by inhibiting NF-κB (Liu et al. 2015b). Similarly, RCAN1 suppressed viability of glioma cells and induced glioma cell apoptosis by inhibiting the NF-κB pathway (Chen et al. 2017). Kaposi’s sarcoma (KS), which is caused by KS-associated herpesvirus (KSHV), is a multifocal angio-proliferative neoplasm. The K15 protein, a KSHV protein, results in KSHV-induced angiogenesis, which is brought about by activation of CN-NFAT1-dependent RCAN1 expression via PLCc1 (Barin et al. 1995; Cai et al. 2010; Ganem 2010).

Above all, RCAN1 has been shown to play diverse roles in regulating cancer development in different organs. Except for hypopharyngeal and laryngopharynx cancer, RCAN1 (or RCAN1.4) inhibits the tumorigenesis and progression of solid organ tumors by inhibiting the CN pathway. In lymphoma and glioma, RCAN1 inhibits the NF-κB pathway. And in hematologic tumor, RCAN1.1 increases drug sensitivity (Table 2).

## RCAN1 and cancer therapy

### Associations between RCAN1, prognosis, and clinical features

Data from the TCGA database show that high levels of RCAN1 expression are strongly correlated to longer overall survival in cases of kidney renal clear cell carcinoma (KIRC), KIRP, and LIHC, and high RCAN1 expression levels are correlated to poorer overall survival in cases of brain lower grade glioma (LGG), mesothelioma (MESO), and thyroid carcinoma (THCA) (Fig. 4B). In addition, RCAN1 expression is associated with human cancer grade and stage. Increased RCAN1 expression is associated with a lower tumor stage in cervical squamous cell carcinoma and endocervical adenocarcinoma (CESC), head and neck squamous cell carcinoma (HNSC), KIRC, and LIHC and with a higher tumor grade in LGG (Fig. 4C). The increased RCAN1 expression is associated with a lower tumor stage in KIRC, KIRP, LIHC, THCA, and uterine corpus endometrial carcinoma (UCEC) and a higher tumor stage in BLCA (Fig. 4D).

In breast cancer, primary HCC, PDAC, colon cancer, ccRCC, and bladder cancer, RCAN1 or RCAN1.4 shows considerably low levels of expression in cancer tissues and results in a poorer prognosis (Jin et al. 2017; Lao et al. 2021; Deng et al. 2020) (Sebio et al. 2015) (Song et al. 2018; Eissa et al. 2019). RCAN1.4 downregulation is also correlated with higher tumor stage, poor tumor differentiation, bigger tumor size, and vascular involvement in HCC (Lao et al. 2021). Increased levels of serum RCAN1 were associated with a better prognosis of PDAC patients (Jin et al. 2017). In CRC, RCAN1 is regarded as a biomarker to predict recurrence in stages II and III of colon cancer (Sebio et al. 2015). In patients with ccRCC, RCAN1 mRNA levels may be a diagnostic biomarker (Song et al. 2018). The levels of RCAN1 in urine samples of the bladder cancer group were lower than those in the control group. The diagnostic value of RCAN1 levels was higher than negative cytology—a common noninvasive diagnostic method for bladder cancer (Eissa et al. 2019). In addition, significantly higher RCAN1.4 expression was found in well-differentiated endometrial adenocarcinoma than in normal endometrium, and its expression decreased with tumor grade (Sales et al. 2010). In contrast, in laryngopharynx cancer and hypopharyngeal cancer with poorly differentiated pathological degree and advanced TNM staging, the positive rates of RCAN1 expression were high (Lü et al. 2011). In brief, the expression of RCAN1 may be regarded as an important biomarker in judging malignancies, distant metastases, and prognoses.

### Therapeutic potential of RCAN1 in different cancers

Considering its pivotal roles in cancer pathogenesis, targeting RCAN1 and the CN-NFAT pathway may offer a potential therapeutic target for cancer treatment. To date, no specific drug has been developed to target RCAN1. However, the good news is that Sun et al. discovered a new RCAN1 aptamer (RCAN1-s14) that was useful for preparing drugs for targeted inhibition of RCAN1 protein and treatment of e.g., Down syndrome, neurodegenerative diseases, inflammatory diseases, and cancer (Sun et al. 2022). The regulatory effect of RCAN1 on CN activity is bidirectional and is dependent on its expression level and phosphorylation status. In other words, the expression of RCAN1 in different cancers can have different roles in tumor progression. Further, the effects of different isoforms of RCAN1 remain unclear. It is reported that RCAN1 has carcinogenesis and angiogenesis effects in some cancers (Lv et al. 2017; Bala et al. 2012; Lu et al. 2011). Meanwhile, RCAN1 plays both beneficial and detrimental roles in the pathogenesis of various diseases, such as various cardiovascular diseases (Wang et al. 2020; Roy-Vallejo et al. 2020; Torac et al. 2014), and Alzheimer’s disease (Wu et al. 2014; Fu and Wu 2018). Thus, although targeting RCAN1 expression may have therapeutic potential for cancers, there are many challenges that should be considered.

Pharmacological CN inhibitors (CNI), including tacrolimus (FK506) and cyclosporin A (CsA), are commonly used as immunosuppressive drugs in patients who have undergone organ transplantation and those with autoimmune disorders. CNI prevents CN-mediated NFAT dephosphorylation by binding to and blocking the PxIxIT- and LxVP-binding regions of CN, which is an RCAN1-inhibitory mechanism in the CN-NFAT pathway (Lü et al. 2011; Bercovich et al. 2008). CNI has a bidirectional role in various tumors. In breast cancer, tacrolimus has been found to inhibit angiogenesis and breast carcinoma growth (Zhao et al. 2016; Siamakpour-Reihani et al. 2011). In colorectal cancer, CsA inhibits tumor growth as a result of lower levels of c-Myc, p21(WAF1/CIP1), and proliferating cell nuclear antigen (PCNA) (Masuo et al. 2009; Werneck et al. 2012). Preclinical studies show that the tumor inhibitory mechanism of CsA involves the sensitization of lung cancer cells to crizotinib via suppression of the Ca2 + /CN/Erk pathway. Treatment with a combination of CsA and crizotinib might have potential in MET-amplified lung cancer (Liu et al. 2019). CsA increases pancreatic cancer cell response to phospho-sulindac (P-S) treatment by overcoming NFATc1-mediated resistance (Murray et al. 2014). In MDSL cells, cyclosporin increases sensitivity to LEN in MDS/AML (He et al. 2020). However, CNI can increase the risk of aggressive cancer progression (Carenco et al. 2015; Hendrikx et al. 2019). For example, CNI increases the rate of HCC recurrence after liver transplantation (Vivarelli et al. 2010, 2008), as well as the risk of RCC in transplant patients (Balan et al. 2017). Therefore, it is crucial to examine the anti-cancer effects of RCAN1 when it is combined with other anti-tumor drugs. However, severe side effects caused by CNI limit its clinical use.

## Discussion and conclusions

We now recognize that RCAN1 is a potent endogenous inhibitor of CN, which plays crucial roles in cancer. In most cancers, except laryngopharynx and hypopharyngeal cancer, RCAN1 expression suppresses cell proliferation, migration, invasion, and angiogenesis, and promotes cell apoptosis by inhibiting CN-NFAT.

The functions of RCAN1 in the tumor immune microenvironment are still unknown. Data from the TCGA database show that the expression of RCAN1, as well as some chemokines and their receptors, correlate positively in most cancers (Fig. 4E, F). Moreover, most immune checkpoint inhibitors (immunoinhibitors) and major histocompatibility complex molecules are significantly and positively correlated with RCAN1, such as CD274, IL-10, KDR, and PDCD1LG2 (Fig. 4G). RCAN1 expression is also positively correlated with several immunostimulators, especially IL-6 (Fig. 4F). To further elucidate the association between RCAN1 and TILs, we analyzed the association between RCAN1 expression and TILs in human cancers. It is clear that the expression of RCAN1 is significantly positively correlated with the levels of many immune cells, including CD4 + T cells, CD8 + T cells, Tregs, natural killer cells, and so on, in 30 types of cancer (Fig. 4I).

Furthermore, our group found that RCAN1.4 overexpression resulted in a significant decrease in cell resistance against T-cell-mediated tumor cell death under in vitro conditions (Lao et al. 2021). However, RCAN1.4 had an inhibitory effect on the expression of CXCL8, IL-8, and IL-11 (Sales et al. 2009, 2010; Maldonado-Pérez et al. 2009). IL-8, a member of CXC chemokine family, played crucial roles in neutrophil chemotaxis/activation, as well as T-cell chemotaxis (Larsen et al. 1989). Presumably, these results imply that RCAN1 influences the biological functions of tumor cells, and is also important for the interaction between other immune components of the tumor microenvironment. Altogether, the data suggest that further studies are needed to understand the role and mechanisms of RCAN1 in the tumor immune microenvironment, and RCAN1 merits further investigation as an attractive next-generation immune candidate cancer target.

At present, increasing evidence have found that RCAN1 can inhibit tumors in most cancers. Consequently, we urgently need to develop a specific drug targeting RCAN1 and assess this drug in clinical trials. Therefore, further investigations should focus on (1) elucidating the detailed mechanisms via which RCAN1 regulates tumor cell function; (2) explore the function of RCAN1 in the tumor microenvironment; and (3) target RCAN1 expression to develop a specific drug and to confirm its therapeutic potential for cancer. Although many unanswered questions remain, and extensive preclinical validation is warranted, we hope that agents targeting RCAN1 can lead to a new antitumor approach to provide effective cancer therapeutics.

## Data Availability

Not applicable.

## Abbreviations

HSA21: Trisomy of chromosome 21
DSCR1: Down syndrome critical region 1
CN: Calcineurin
RCAN1: Regulator of calcineurin 1
Ser: Serine
Thr: Threonine
NFAT: Nuclear factor of activated T cell
RRM: RNA-recognition motif
SLiMs: Short linear motifs
pRCAN1: Phosphorylated RCAN1
IGF-1: Insulin-like growth factor 1
IFI27: Interferon alpha inducible protein 27
CXCL8: C-X-C motif chemokine ligand 8
IL-8: Interleukin-8
IL-11: Interleukin-11
VEGFA: Vascular endothelial growth factor A
LEN: Lenalidomide
GC: Glucocorticoid
GREs: Glucocorticoid response elements
HG: High glucose
NFE2L3: Erythroid 2-like 3
ccRCC: Clear cell renal cell carcinoma
SEs: Super-enhancers
GSK-3β: Glycogen synthase kinase 3β
MAPK: Mitogen-activated protein kinase
DYRK1A: Dual specificity tyrosine-phosphorylation-regulated kinase 1A
TAK1: TGF-β activated kinase 1
TAB: TAK1 binding protein
MEKK3: Mitogen-activated protein kinase kinase 3
MEK5: Mitogen-activated protein kinase kinase 5
BMK1: Big mitogen-activated protein kinase 1
NIK: Mitogen-activated protein kinase kinase kinase 14
PKA: Protein kinase A
TCGA: The Cancer Genome Atlas
BLCA: Bladder urothelial carcinoma
BRCA: Breast invasive carcinoma
KICH: Kidney chromophobe
KIRP: Kidney renal papillary cell carcinoma
LIHC: Liver hepatocellular carcinoma
LUAD: Lung adenocarcinoma
DLBC: Lymphoid neoplasm diffuse large B-cell lymphoma
GBM: Glioblastoma multiforme
PAAD: Pancreatic adenocarcinoma
SKCM: Skin cutaneous melanoma
THYM: Thymoma
SCLC: Small cell lung cancer
As2O3: Arsenic trioxide
NSCLC: Non-small cell lung cancer
HCC: Hepatocellular carcinoma
miRNA: MicroRNA
DEN: Diethylnitrosamine
ENU: Ethylnitrosourea
PanIN: Pancreatic intraepithelial neoplasia
CRC: Colon cancer
PPARγ: Peroxisome proliferator-activated receptor γ
RCC: Renal cell carcinoma
EMT: Epithelial–mesenchymal transition
HIF2α: Hypoxia inducible factor 2 alpha
chemokines: Chemoattractant cytokines
COX: Cyclooxygenase
PGs: Prostaglandins
FP: F-prostanoid
PGF_2α_-FP: Prostaglandin F_2α_-F-prostanoid
PROK1: Prokineticin 1
OSCC: Oral squamous cell carcinoma
MVD: Microvessel density
JAK2: Janus kinase 2
GR: GC receptor
CREB: CAMP response element binding protein
MM: Multiple myeloma
MDS: Myelodysplastic syndromes
AML: Acute myeloid leukemia
Cys: Cyclosporine
KS: Kaposi’s sarcoma
KSHV: KS-associated herpesvirus
KIRC: Kidney renal clear cell carcinoma
LGG: Lower grade glioma
MESO: Mesothelioma
THCA: Thyroid carcinoma
CESC: Cervical squamous cell carcinoma and endocervical adenocarcinoma
HNSC: Head and neck squamous cell carcinoma
UCEC: Uterine corpus endometrial carcinoma
CNI: CN inhibitors
CsA: Cyclosporin A
P-S: Phospho-sulindac
MHC: Major histocompatibility complex
